# Draft Genome Sequence of *Bacillus pumilus* 7P, Isolated from the Soil of the Tatarstan Republic, Russia

**DOI:** 10.1128/genomeA.00599-14

**Published:** 2014-06-12

**Authors:** Elena I. Shagimardanova, Anna A. Toymentseva, Nelly P. Balaban, Ayslu M. Mardanova, Yulia V. Danilova, Oleg A. Gusev, Elena Kostryukova, Irina Karpova, Aleksandr Manolov, Dmitriy Alexeev, Margarita R. Sharipova

**Affiliations:** a Institute of Fundamental Medicine and Biology, Kazan (Volga Region) Federal University, Kazan, Russia; b Scientific Research Institute of Physical-Chemical Medicine, Moscow, Russia

## Abstract

Here, we present a draft genome sequence of *Bacillus pumilus* strain 7P. This strain was isolated from soil as an extracellular RNase-producing microorganism. The RNase of *B. pumilus* 7P is considered to be a potential antiviral and therapeutic antitumor agent, and it might be appropriate for agriculture and academic synthesis of oligoribonucleotides.

## GENOME ANNOUNCEMENT

*Bacillus pumilus* 7P (16S rRNA GenBank accession no. JX129390.1), a Gram-positive endospore-forming aerobic bacterium, was isolated from a soil sample in Russia. This strain is widely used for studying secreted RNases, including enzymes of interest for medical, academic, and industrial utilization (1–4). The *B. pumilus* 7P RNase gene was cloned and sequenced (GenBank accession no. X53697) (5), the corresponding protein was purified, and numerous biological effects were demonstrated (2, 3).

Whole-genome shotgun sequencing was performed using a combination of 454 GS Junior Roche pyrosequencing and the 200-bp chemistry Ion Torrent PGM platform, which provided 43-fold overall genome coverage. The reads were corrected with the SAET 3 tool and assembled with Newbler 2.6. The obtained genome sequence included 33 contigs (>200 bp in size), with a calculated genome size of 3,582,806 bp and a G+C content of 42 mol%.

Genome assemblies for eight strains of *B. pumilus* are already available at GenBank, including one type strain (ATCC 7061), one strain with highly UV-resistant spores (SAFR-032) (6), one alkaline serine protease-producing strain (BA06) (7), one strain with plant growth-promoting activity (INR7), and one strain of industrial and biotechnological interest (CCMA-560). We used them for a comparison and calculated the average nucleotide identities (ANI) using JSpecies (8). Strain 7P was found to be most closely related to strain ATCC 7061 (ANI, 94.6%), followed by strain SAFR-032 (ANI, 94.55%) and strain CCMA-560 (ANI, 91.99%).

The genome was annotated using the NCBI Prokaryotic Genome Annotation Pipeline (PGAAP) (http://www.ncbi.nlm.nih.gov/genomes/static/Pipeline.html). A total of 3,596 genes and 3,460 coding sequences (CDS) were predicted, which is similar to other strains of the same species. The numbers of genes encoding components of amino acid metabolism, carbohydrate metabolism, cell wall and capsule, sporulation, prophage elements, resistance to antibiotics, and toxic compounds were higher in the 7P genome than in the SAFR-032 genome. Seventy-two genes encoding tRNAs and 4 genes encoding rRNAs were detected (5S, 16S, and 23S). The presence of partitioning gene *parA* on contig 13 with 10-fold increased coverage and its circular structure as supposed by the Newbler output suggest that the contig represents a plasmid.

The genome harbors the genes encoding RNase (binase), which is in agreement with previously published data (4, 5). Information about the various genes involved in the pathways related to hydrolase biosynthesis will improve our understanding of the regulatory mechanisms of the production of industrially important and medically relevant enzymes.

### Nucleotide sequence accession number.

The *B. pumilus* 7P whole-genome sequence and annotation data have been deposited at DDBJ/EMBL/GenBank under the accession no. JHUD00000000. The version described in this paper is the first version.
